# Urodynamic effectiveness of a beta-3 adrenoreceptor agonist (vibegron) for a pediatric patient with anticholinergic-resistant neurogenic detrusor overactivity: a case report

**DOI:** 10.1186/s13256-020-02564-w

**Published:** 2021-02-18

**Authors:** Taiki Kato, Kentaro Mizuno, Hidenori Nishio, Takahiro Yasui, Yutaro Hayashi

**Affiliations:** 1 Department of Nephro-urology, Nagoya City University Graduate School of Medical Sciences, Nagoya, Japan; 2 Department of Pediatric Urology, Nagoya City University Graduate School of Medical Sciences, 1 Kawasumi, Mizuho-cho, Mizuho-ku, Nagoya, 467-8601 Japan

**Keywords:** Beta-3 adrenoreceptor agonist, Vibegron, Urodynamics, Neurogenic bladder, Spina bifida

## Abstract

**Background:**

Myelomeningocele, which causes a neurogenic bladder, is usually treated with anticholinergics in children with neurogenic detrusor overactivity (NDO); however, anticholinergics cause side effects such as dry mouth, constipation, attention deficit, and inadequate reduction in detrusor leak point pressure. Vibegron, a novel selective beta-3 adrenoreceptor agonist, is a well-established alternative to anticholinergics in adults with an overactive bladder. It remains unknown whether this agent can be used for pediatric patients. We report the case of a girl with anticholinergic-resistant NDO due to tethered cord syndrome after myelomeningocele repair, who was treated with vibegron.

**Case presentation:**

A 4-year-old Filipino girl had increased frequency of daytime urinary incontinence and foul-smelling urine since the age of 3. Clinical examination revealed constipation, and urinalysis revealed bacteriuria. Voiding cystourethrography revealed an enlarged and trabeculated bladder without vesicoureteral reflux. On the urodynamic study (UDS), she was found to have detrusor overactivity (DO) and low bladder compliance. She could not void and was diagnosed with overflow incontinence. Clean intermittent catheterization (CIC) and orally administered propiverine (0.8 mg/kg/day) were initiated, and urinary incontinence was resolved. She underwent a UDS annually; the UDS at 6 years of age still revealed DO and low bladder compliance in spite of receiving propiverine. The treatment was switched from propiverine to vibegron (1.4 mg/kg/day). On the UDS after a 5-week treatment schedule of vibegron, the DO disappeared and the bladder compliance improved. CIC and orally administered vibegron have been continued for 7 months so far, and she has had no urinary tract infection with no drug-related adverse events.

**Conclusions:**

Vibegron was effective and well tolerated in the treatment of a pediatric patient with NDO. Vibegron improved the urodynamic parameters for anticholinergic-resistant neurogenic bladder. This agent can be a beneficial and preferable alternative therapeutic agent to anticholinergics in patients with anticholinergic-resistant NDO.

## Background

Myelomeningocele is the most common form of spinal dysraphism and the most common primary cause of neurogenic bladder. The combination of clean intermittent catheterization (CIC) and oral anticholinergics has been considered as the standard treatment for children with neurogenic detrusor overactivity (NDO), which can cause upper urinary tract damage due to high bladder pressure [1]. Anticholinergics are effective for the treatment of children with NDO; however, there are some concerns about anticholinergic side effects such as dry mouth, constipation, attention deficit, and inadequate reduction of the detrusor leak point pressure. Thus, some patients need to undergo more invasive procedures [2, 3]. A new, highly selective beta-3 adrenoceptor agonist, vibegron, has recently been introduced for the treatment of patients with overactive bladder (OAB), and has demonstrated significant improvement in micturition, urgency and incontinence episodes [4], and its long-term safety and tolerance [5].

Herein, we report the case of a girl with anticholinergic-resistant NDO due to tethered cord syndrome after myelomeningocele repair, who had recurrent urinary tract infections (UTIs), low bladder compliance, and detrusor overactivity, using urodynamic study. Therefore, she was treated with vibegron.

## Case presentation

**Fig. 1:**
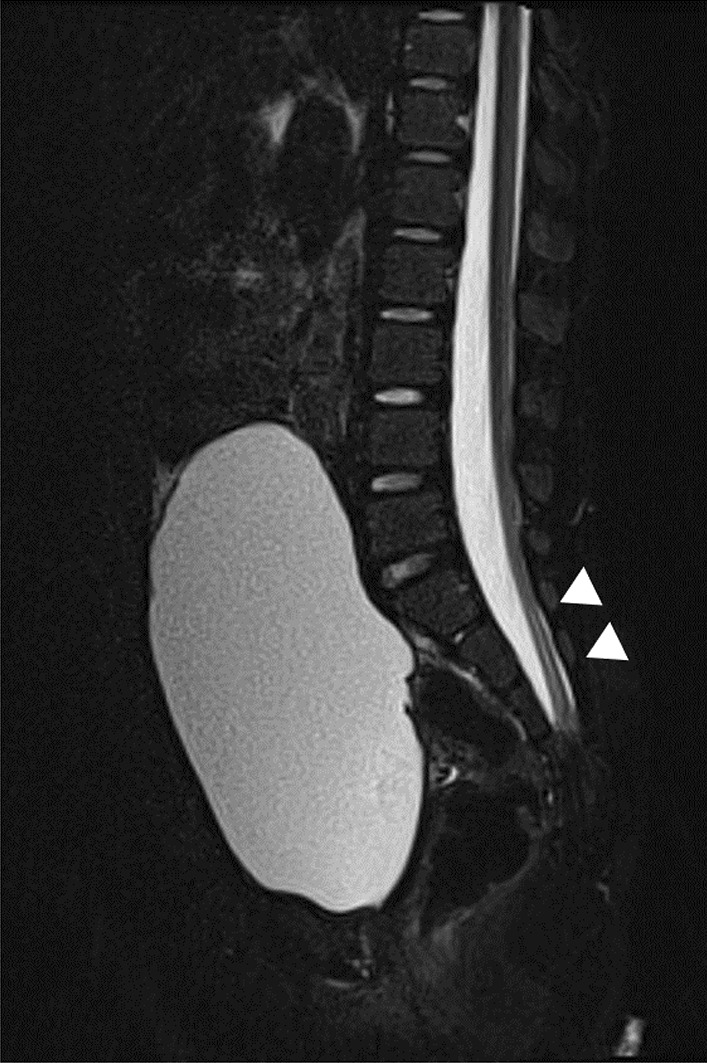
Magnetic resonance imaging of the synsacrum during the first visit. Sagittal T2-weighted image showing tethered spinal cord syndrome and syringomyelia (arrowheads)

**Fig. 2:**
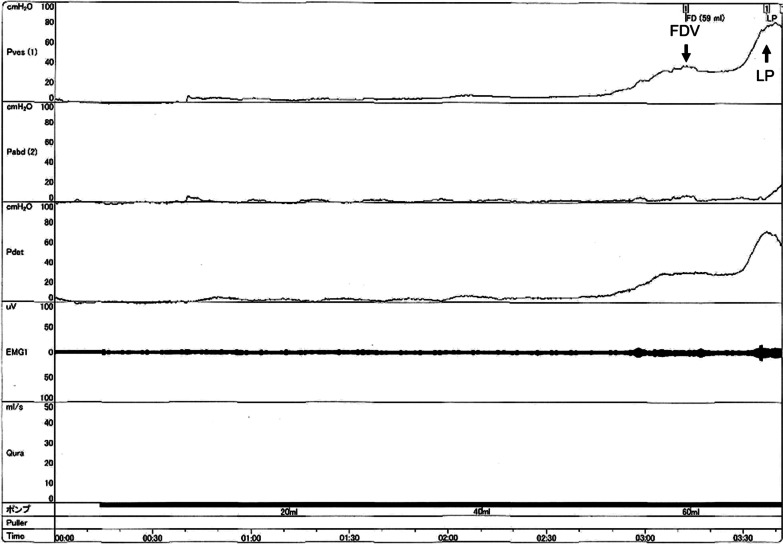
Urodynamic study during the first visit at our hospital. From top to bottom: bladder pressure (cmH_2_O), intra-abdominal pressure (cmH_2_O), detrusor pressure (cmH_2_O), electromyography (μV), and flowmetry (mL/second). Horizontal scales: bladder volume (mL) and time (seconds). *FDV* first desire to void, *LP* leak point

**Fig. 3:**
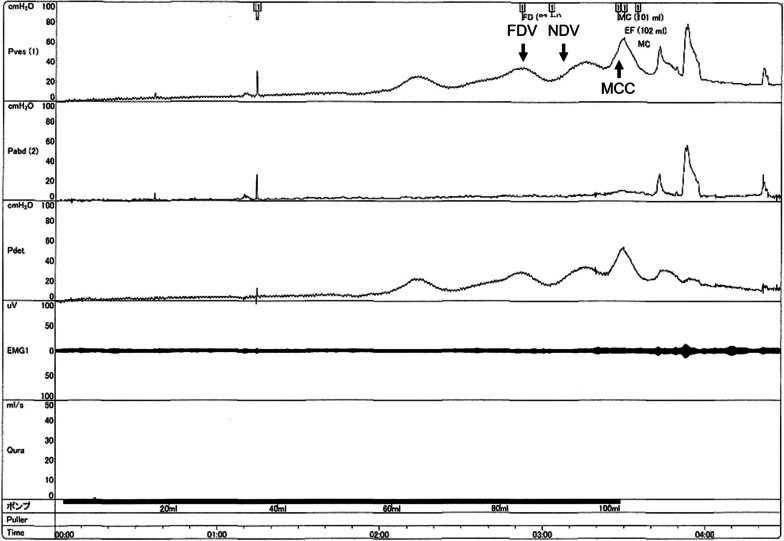
Urodynamic study during treatment with oral propiverine. *FDV* first desire to void, *NDV* normal desire to void, *MCC* maximum cystometric capacity

**Fig. 4:**
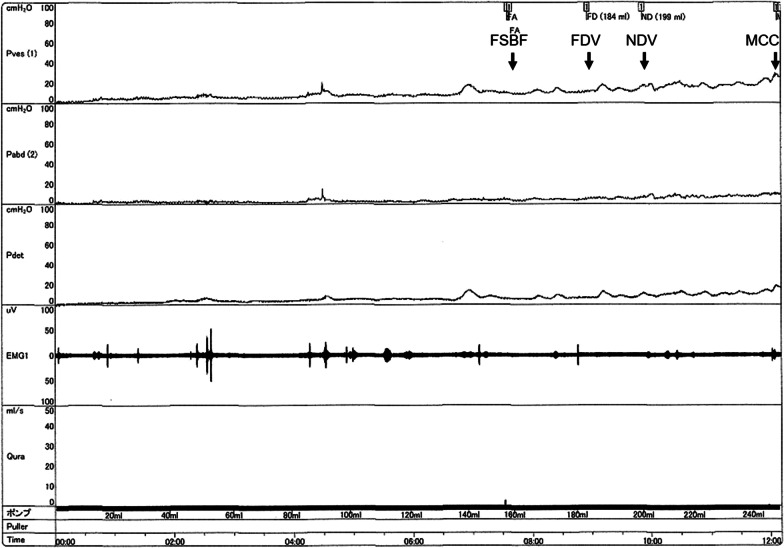
Urodynamic study during treatment with orally administered vibegron after changing from propiverine. *FSBF* first sensation of bladder filling, *FDV* first desire to void, *NDV* normal desire to void, *MCC* maximum cystometric capacity

A 4-year-old Filipino girl was referred to our hospital due to increased frequency of daytime urinary incontinence and foul-smelling urine since the age of 3. She was diagnosed with a lumbosacral myelomeningocele at 21 weeks of pregnancy and born to non-consanguineous parents at 37 weeks of gestation, weighing 2908 g. She underwent myelomeningocele repair 2 days after birth and did not have hydrocephalus. She was able to walk unaided 11 months after birth. Although she had a history of recurrent UTIs, she did not visit a urologist office regularly after surgery. At her first visit to our hospital, her physical and neurological examination was normal, that is, she had a maximum Glasgow Coma Scale score, steady gait, and no paralysis in her legs, and could run as much as other healthy children of the same age. She had a normal bilateral reflex knee jerk and Achilles tendon reflex. Clinical examination revealed constipation and no flexion contractures of her legs. Urinalysis revealed bacteriuria. Magnetic resonance imaging (MRI) revealed a tethered spinal cord (TSC) and syrinx at the S2–3 levels (Fig. 1). A pediatric voiding cystourethrography revealed an enlarged and trabeculated bladder without any vesicoureteral reflux. A urodynamic study (UDS) showed detrusor overactivity and low bladder compliance (1.8 mL/cmH_2_O). The maximum cystometric bladder capacity was 67 mL, with no specific bladder sensation, and the detrusor leak-point pressure was 77 cmH_2_O. She was unable to void. She was diagnosed with overflow incontinence (Fig. 2). Clean intermittent catheterization (CIC) was performed and 10 mg of propiverine (0.8 mg/kg body weight/day) was administered orally, and her urinary incontinence resolved. She underwent repeated UDS annually. A UDS at 6 years of age (she had been receiving propiverine for 2 years and 5 months) showed detrusor overactivity and low bladder compliance (3.7 mL/cmH_2_O), in spite of receiving 10 mg of orally administered propiverine per day. The maximum cystometric bladder capacity was 101 mL, and detrusor leak-point pressure was 50 cmH_2_O (Fig. 3). She had no urinary incontinence or UTI. However, the cystometrogram showed that her bladder pressure was persistently high. To reduce pressure on the urinary bladder, and avoid the risk of upper urinary tract damage, treatment was switched from propiverine to vibegron, a beta-3 adrenoceptor agonist. After obtaining the patient’s and parental informed consent, a single daily dose of 25 mg of vibegron (Beova; Kissei Pharmaceutical Co., Ltd., Tokyo, Japan) (1.4 mg/kg/day) was administered. After 5 weeks of once-daily vibegron treatment, UDS was performed and showed disappearance of detrusor overactivity, increased maximum cystometric bladder capacity (251 mL), and improved bladder compliance (9.0 mL/cmH_2_O) (Fig. 4). She has continued to receive vibegron that has been well tolerated and to perform CIC for 7 months so far, and no UTI, incontinence, or drug-related adverse events have been observed. No abnormalities were found in blood pressure (98/60 mmHg) or heart rate (72 beats/min) or on the blood tests after a month of receiving vibegron. Her detailed laboratory data (before and after taking vibegron) are presented in Table 1.

**Table 1:** Laboratory and urinalysis data findings before and after regimen of orally administered vibegron

Laboratory test	Before vibegron	After vibegron	Reference range	Units
	Results	Results		
WBC	12.5	6.0	3.3–8.6	× 10^3^/µL
RBC	4.54	4.51	3.86–4.92	× 10^6^/µL
Hb	12.7	12.8	11.6–14.8	g/dL
HCT	37.2	38.4	35.1–44.4	%
PLT	248	328	158–348	× 10^3^/µL
TP	7.3	6.9	6.6–8.1	g/dL
ALB	4.4	4.8	4.1–5.1	g/dL
AST	28	23	13–30	IU/L
ALT	12	13	7–23	IU/L
LDH	228	216	124–222	IU/L
ALP	469	706	106–322	IU/L
CRE	0.34	0.39	0.46–0.79	mg/dL
BUN	8.6	11.1	8–20	mg/dL
UA	6.1	2.9	2.6–5.5	mg/dL
Na	138	138	138–145	mmol/L
K	3.8	4.2	3.6–4.8	mmol/L
CL	100	103	101–108	mmol/L
Ca	9.9	9.6	8.8–10.1	mg/dL

Laboratory test	Before vibegron	After vibegron	Reference range	Units
	Results	Results		
Urinalysis				
Color	Yellow	Yellow	Yellow	
Clarity	Cloudy	Cloudy	Clear	
Specific gravity	1.017	1.021	1.003–1.030	
pH	7.5	8.0	4.5–8.0	
Urobilinogen	Normal	Normal	Normal	
Bilirubin	−	−	Negative	
Leukocytes	3+	1+	Negative	
Nitrite	+	+	Negative	
Blood	−	−	Negative	
Protein	+−	−	Negative	
Glucose	−	−	Negative	
Ketone	−	−	Negative	
Urine microscopy				
Red blood cells	5–9	1–4	Negative	/hpf
White blood cells	50–99	10–19	Negative	/hpf
Casts	−	−	Negative	/hpf
Dysmorphic RBC	Negative	Negative	Negative	
Epithelial cells	Negative	Negative	Negative	/hpf
Bacteria	+	+	Negative	
Urine culture	*E. coli*	*E. coli*	Negative	

Before: while continuing to take propiverine. After: after taking vibegron for a month

*ALB* albumin, *ALP* alkaline phosphatase, *ALT* alanine transaminase, *AST* aspartate aminotransferase, *BUN* blood urea nitrogen, *Ca* calcium, *CL* chloride, *CRE* creatinine, *Hb* hemoglobin, *HCT* hematocrit, *LDH* lactate dehydrogenase, *K* potassium, *Na* sodium, *PLT* platelets, *RBC* red blood cells, *TP* total protein, *UA* uric acid, *WBC* white blood cells

## Discussion and conclusions

To the best of our knowledge, this is the first report to show that vibegron can improve high bladder pressure using urodynamic parameters in a child with anticholinergic-resistant NDO due to a TSC after surgery of myelomeningocele.

Secondary TSC occurs in 3–30% of patients with a history of spinal cord dysraphism repair. The symptoms of TSC include back pain, leg weakness, foot deformity, and lower urinary tract dysfunction such as urinary incontinence or retention [6]. It is often difficult to diagnose secondary TSC in patients with a prior history of spinal cord dysraphism repair, especially if neurological symptoms are not evident and there is no typical urological dysfunction. Urodynamic evaluations are important for diagnosis and subsequent management, because the urinary symptoms and urodynamic parameters may not always match [7]. Although several reports [6, 8] have noted that lower urinary tract symptoms and urodynamic parameters improve by untethering surgery for secondary TSC, these were retrospective studies with a small number of patients, and some patients had inadequate improvement in low bladder compliance on UDS; thus, the level of evidence can be considered weak. The patient in the current report is in follow-up with the neurosurgeon and undergoes synsacrum MRI scans regularly; however, she and her parents have declined untethering surgery, as she has no symptoms, and untethering surgery is not a riskless procedure.

The primary principles in the management of NDO include the preservation of renal function, along with avoidance of UTIs by maintaining low bladder pressure. Anticholinergic agents are the first-line treatment for NDO; however, their use may be limited by their side effects such as dryness of the mouth, dizziness, and headache [2]. Mirabegron, the first beta-3 adrenoreceptor agonist, was recently reported to improve incontinence episodes and urodynamic parameters for neurogenic bladder in pediatric patients with spina bifida [9]. Mirabegron has been approved for use in treating OAB symptoms and as an anticholinergic agent. Beta-3 adrenoreceptor agonists induce relaxation of the detrusor muscle, resulting in decreased frequency of micturition and improvement in urinary continence [10]. Mirabegron has an effect similar to an anticholinergic agent, with the benefit of significantly fewer side effects such as dry mouth and constipation [11]. Moreover, mirabegron has been reported to be effective for patients with a neurogenic bladder unresponsive to anticholinergic agents [12, 13]. However, mirabegron was found to induce reproductive toxicity and was associated with atrophy of the reproductive organs in rats, and thus should be avoided in children. Mirabegron is also known to inhibit CYP2D6, a cytochrome P450 enzyme, and is therefore a potential source of drug–drug interactions [5].

Vibegron is a novel, potent, and highly selective beta-3 adrenoreceptor agonist without reproductive side effects; it is effective, well tolerated, and safe for the treatment of adult patients with OAB [4, 5]. Regarding drug metabolism, vibegron showed no induction or inhibitory effect on cytochrome P450 enzymes, suggesting a low risk of drug–drug interactions [5]. It was also recently reported that vibegron is useful and safe for the pediatric population with treatment-resistant monosymptomatic nocturnal enuresis [14].

In this case, our patient received vibegron because her high bladder pressure did not improve with the use of oral anticholinergic agents. She has continued CIC and has been receiving vibegron for 7 months, and she has no incontinence, no UTIs, and no drug adverse events so far. No abnormalities were found in blood pressure or heart rate or on the blood tests after a month of receiving vibegron.

The exact reason for the effectiveness of beta-3 adrenoceptor agonists as an alternative to anticholinergics is unknown. The pharmacological action of anticholinergics and beta-3 adrenoceptor agonists is different. The effect of urinary bladder body relaxation in the present case may be stronger with the use of beta-3 adrenoreceptor agonists than with anticholinergics. There is evidence in humans that sacral decentralization results in a change in the detrusor muscle adrenergic receptor response; parasympathetic sacral nerve injury can reverse the usual beta-adrenergic receptor response (smooth muscle relaxation) to an alpha-adrenergic receptor effect (smooth muscle contraction) [15]; thus, stimulation of beta-adrenergic receptors may induce relaxation of the bladder body, resulting in increased bladder compliance, maximum cystometric bladder capacity, and inhibition of detrusor overactivity.

In conclusion, vibegron improved the urodynamic parameters in our patient with anticholinergic-resistant neurogenic bladder. Vibegron might be an alternative agent for pediatric patients with neurogenic bladder. Further studies are needed to evaluate the efficacy and safety of vibegron in the pediatric population.

## Data Availability

Data sharing is not applicable to this article as no datasets were generated during the current study.

## Abbreviations

NDO: Neurogenic detrusor overactivity
DO: Detrusor overactivity
UDS: Urodynamic study
CIC: Clean intermittent catheterization
OAB: Overactive bladder
UTI: Urinary tract infection
MRI: Magnetic resonance imaging
TSC: Tethered spinal cord

## References

[1] Bauer SB. The management of the myelodysplastic child: a paradigm shift. BJU Int. 2003;92(Suppl 1):23–8. 10.1046/j.1464-410x.92.s1.2.x 12969005

[2] Ferrara P, D’Aleo CM, Tarquini E, Salvatore S, Salvaggio E. Side-effects of oral or intravesical oxybutynin chloride in children with spina bifida. BJU Int. 2001;87:674–8. 10.1046/j.1464-410x.2001.02152.x 11350411

[3] Schulte-Baukloh H, Murtz G, Heine G, Austin P, Miller K, Michael T, *et al.* Urodynamic effects of propiverine in children and adolescents with neurogenic bladder: results of a prospective long-term study. J Pediatr Urol. 2012;8:386–92. 10.1016/j.jpurol.2011.07.014 21907623

[4] Yoshida M, Takeda M, Gotoh M, Nagai S, Kurose T. Vibegron, a novel potent and selective β(3)-adrenoreceptor agonist, for the treatment of patients with overactive bladder: a randomized, double-blind, placebo-controlled phase 3 study. Eur Urol. 2018;73:783–90. 10.1016/j.eururo.2017.12.022 29366513

[5] Yoshida M, Kakizaki H, Takahashi S, Nagai S, Kurose T. Long-term safety and efficacy of the novel beta3-adrenoreceptor agonist vibegron in Japanese patients with overactive bladder: a Phase III prospective study. Int J Urol. 2018;25:668–75. 10.1111/iju.13596 29752752

[6] Alzahrani A, Alsowayan O, Farmer JP, Capolicchio JP, Jednak R, El-Sherbiny M. Comprehensive analysis of the clinical and urodynamic outcomes of secondary tethered spinal cord before and after spinal cord untethering. J Pediatr Urol. 2016;12(101):e1-6. 10.1016/j.jpurol.2015.08.011 26454453

[7] Yener S, Thomas DT, Hicdonmez T, Dagcinar A, Bayri Y, Kaynak A, *et al.* The effect of untethering on urologic symptoms and urodynamic parameters in children with primary tethered cord syndrome. Urology. 2015;85:221–6. 10.1016/j.urology.2014.10.008 25530386

[8] Geyik M, Geyik S, Şen H, Pusat S, Alptekin M, Yılmaz AE, *et al.* Urodynamic outcomes of detethering in children: experience with 46 pediatric patients. Childs Nerv Syst. 2016;32:1079–84. 10.1007/s00381-016-3053-y 26969174

[9] Park JS, Lee YS, Lee CN, Kim SH, Kim SW, Han SW. Efficacy and safety of mirabegron, a β3-adrenoceptor agonist, for treating neurogenic bladder in pediatric patients with spina bifida: a retrospective pilot study. World J Urol. 2019;37:1665–70. 10.1007/s00345-018-2576-0 30511212

[10] Yamaguchi O, Marui E, Igawa Y, Takeda M, Nishizawa O, Ikeda Y, *et al.* Efficacy and safety of the selective β3-adrenoceptor agonist mirabegron in Japanese patients with overactive bladder: a randomized, double-blind, placebo-controlled, dose-finding study. Lower Urinary Tract Symptoms. 2015;7:84–92. 10.1111/luts.12053 26663687

[11] Kelleher C, Hakimi Z, Zur R, Siddiqui E, Maman K, Aballea S, *et al.* Efficacy and tolerability of mirabegron compared with antimuscarinic monotherapy or combination therapies for overactive bladder: a systematic review and network meta-analysis. Eur Urol. 2018;74:324–33. 10.1016/j.eururo.2018.03.020 29699858

[12] El Helou E, Labaki C, Chebel R, El Helou J, Abi Tayeh G, Jalkh G, *et al.* The use of mirabegron in neurogenic bladder: a systematic review. World J Urol. 2019. 10.1007/s00345-019-03040-x. 31802206

[13] Kamei J, Furuta A, Akiyama Y, Niimi A, Ichihara K, Fujimura T, *et al.* Video-urodynamic effects of mirabegron, a β3-adrenoceptor agonist, in patients with low-compliance bladder. Int J Urol. 2015;22:956–61. 10.1111/iju.12867 26177781

[14] Fujinaga S, Watanabe Y, Nakagawa M. Efficacy of the novel selective beta3-adrenoreceptor agonist vibegron for treatment-resistant monosymptomatic nocturnal enuresis in children. Int J Urol. 2020;27:693–4. 10.1111/iju.14264 32430925

[15] Sundin T, Dahlström A, Norlén L, Svedmyr N. The sympathetic innervation and adrenoreceptor function of the human lower urinary tract in the normal state and after parasympathetic denervation. Invest Urol. 1977;14:322–8. 836469

